# Networking of the Human Cerebellum: From Anatomo-Functional Development to Neurosurgical Implications

**DOI:** 10.3389/fneur.2022.806298

**Published:** 2022-02-04

**Authors:** Alessandro De Benedictis, Maria Camilla Rossi-Espagnet, Luca de Palma, Andrea Carai, Carlo Efisio Marras

**Affiliations:** ^1^Neurosurgery Unit, Department of Neurosciences, Bambino Gesù Children's Hospital, Istituto di Ricovero e Cura a Carattere Scientifico, Rome, Italy; ^2^Neuroradiology Unit, Imaging Department, Bambino Gesù Children's Hospital, Istituto di Ricovero e Cura a Carattere Scientifico, Rome, Italy; ^3^Neurology Unit, Department of Neurosciences, Bambino Gesù Children's Hospital, Istituto di Ricovero e Cura a Carattere Scientifico, Rome, Italy

**Keywords:** white matter, cerebellar anatomy, neurosurgery, posterior fossa, structural connectivity

## Abstract

In the past, the cerebellum was considered to be substantially involved in sensory-motor coordination. However, a growing number of neuroanatomical, neuroimaging, clinical and lesion studies have now provided converging evidence on the implication of the cerebellum in a variety of cognitive, affective, social, and behavioral processes as well. These findings suggest a complex anatomo-functional organization of the cerebellum, involving a dense network of cortical territories and reciprocal connections with many supra-tentorial association areas. The final architecture of cerebellar networks results from a complex, highly protracted, and continuous development from childhood to adulthood, leading to integration between short-distance connections and long-range extra-cerebellar circuits. In this review, we summarize the current evidence on the anatomo-functional organization of the cerebellar connectome. We will focus on the maturation process of afferent and efferent neuronal circuitry, and the involvement of these networks in different aspects of neurocognitive processing. The final section will be devoted to identifying possible implications of this knowledge in neurosurgical practice, especially in the case of posterior fossa tumor resection, and to discuss reliable strategies to improve the quality of approaches while reducing postsurgical morbidity.

## Introduction

In the past, the cerebellum was considered to be substantially involved in sensory-motor coordination through a loop circuit between the cerebellar cortex and the motor cortex, passing through the basilar pontine nuclei (descending way) and the thalamus (ascending way) (1, 2).

However, growing evidence coming from neuroanatomic, neuroimaging, physiology, and pathology studies has led to a reassessment of the classic view of a selective cerebro-cerebellar connection system in favor of a more complex organization involving distinct, parallel, and segregated networks constituted by loop-shaped connections between different neuronal subgroups. This structural complexity may explain the role of the cerebellum not only in motor function, but also in a variety of cognitive, affective, social, and behavioral processes as well (3). As for the supratentorial compartment, cerebellar networks undergo intensive development and rearrangement from childhood to adulthood, allowing for maturation of functional processing (4–6).

This process is strictly dependent on the correct building of white matter (WM) tracts, forming the substrate for structural connectivity and reinforcement of functional interactions. A large variety of pathways are needed to ensure a fast, efficient, multidistance and multidirectional integration between cortical and subcortical regions (2, 7). On the other hand, many diseases may reflect alterations affecting the cerebellar WM architecture (8, 9).

Neurosurgeons are frequently involved in approaching the cerebellum, especially for the management of tumors or vascular malformations. Therefore, an accurate awareness of the anatomo-functional organization of the cerebellum is mandatory to improve the quality of surgical results while minimizing postoperative long-term neurological deficits, which still occur at a not negligible frequency according to recently published series (10).

The aim of this review is to summarize the current scientific evidence covering the anatomo-functional properties of the cerebellar connectome. In the first section, we will focus on the maturation process of afferent and efferent neuronal circuitry, and we will describe the involvement of these networks in different aspects of neurocognitive processing. The second section will be devoted to identifying possible implications of this knowledge in neurosurgical practice, especially in case of posterior fossa tumor resection, and to discuss reliable strategies to improve the quality of approaches.

## The Cerebellar Connectome

### History

After early pioneering descriptions, a substantial contribution to research on cerebellar anatomy came from Santiago Ramón y Cajal (1854–1934). By adopting the revolutionary method introduced by Camillo Golgi (1843–1926) of nervous tissue fixation in potassium bichromate followed by submersion in a solution of silver nitrate, Cajal identified all the elements constituting the cerebellar cortex. He provided a precise description of avian Purkinje cells, including their axons course, their collaterals, and the spines of their dendrites. Moreover, he showed the reciprocal relationships of Purkinje cells with other cells and the topography of the cellular elements, their dendrites, climbing and mossy fibers in respects to different cortical layers. In this way, he provided evidence for the network anatomo-functional organization of the cerebellar cortex: “the transmission of the nervous impulse is always from the dendritic branches and the cell body to the axon or functional process” (11–13).

Later, the highly homogenous and compartmentalized structure of the cerebellar cortex, and distribution of afferents fields were further highlighted. In 1940, Jansen and Brodal were the first to demonstrate that the cortico-nuclear outputs are organized according to a definite columnar architecture and mediolateral sagittal orientation. The same topography was recognized also for the afferent pathways by following studies (14).

Thanks to methodological advancement for morphological investigation within experimental animals, including histological tract-tracing studies, degeneration techniques, anterograde and retrograde tract-tracing techniques, and transneuronal tracing techniques, the WM architecture of the cerebellum was further characterized, especially regarding the connectivity patterns with the cerebrum. These methods revealed the circuit organization of cerebro-cerebellar networks, consisting of functionally segregated neuronal subgroups interconnected by specific and distinct WM bundles (15–20).

A revolutionary contribution to the exploration of WM anatomy came from Joseph Klingler (1888–1966), who in 1935 introduced an innovative method for the preparation of *post-mortem* human specimens. This approach, based on fiber separation induced by freeze/defrost formalin-fixation process, allowed for easier visualization and dissection of WM fascicles (21, 22). Moreover, development of tractography technique made possible the *in vivo* quantitative and qualitative characterization of both the physiological and pathological pattern of WM connections. The single or combined use of neuroimaging and dissection analysis opened the door to a more accurate and systematic identification of major intra- and inter-hemispheric WM fascicles and reciprocal relationships, for both scientific and therapeutic purposes (23–31).

Even if less frequently reported in comparison with the supratentorial compartment, *in-vivo* and *ex-vivo* methods were also adopted for investigating cerebellar WM anatomy. The most recent available studies reported detailed exposition and three-dimensional representation of different gray and WM structures, focusing on reciprocal relationships and practical surgical implications, mainly concerning the indications, advantages, limitations, and possible risks of specific approaches (32–36).

### Maturation

The development of the human cerebellum is a highly protracted and orchestrated process, extending from the early first trimester to the end of the second post-natal year. The cerebellum is a derivative of two rhombomeres respectively located caudally (near the tail) and rostrally (near the front) in the alar plate of the neural tube, which develops along the rhombencephalon of the embryonic brain (6).

Between 20 and 40 weeks of gestation, the morphogenesis of the cerebellar cortex includes neuronal proliferation, migration, differentiation, axon growth, synaptogenesis, and pruning. At birth, the human cerebellar cortex has four layers: the external granular layer (EGL), the molecular layer (ML), the Purkinje cell layer (PL), and the internal granular layer (IGL). In a matter of 12–24 months, the number of layers reduces from four to three, by progressive increasing of the ML and the PL in association with gradual disappearance of the EGL thickness, due to decreased proliferation as well as migration of granule neurons into the IGL (4, 37, 38).

The cerebellar hemispheric WM is among the first region of the brain to myelinate. Available data coming from volumetric, epidemiologic and tractography studies showed that the myelination process is particularly high during the third trimester and continues, but less drastically, between 2 and 5 years of age (39). Moreover, cerebellar WM does not myelinate uniformly, but along a temporal gradient starting from the archi-cerebellum and followed by the paleo-cerebellum and the neo-cerebellum (40).

Concerning the development of extracerebellar connectivity, over the last decades both post-mortem microdissection and *in-vivo* tractography reconstructions allowed to characterize the maturation process and relationships with the core of the cerebellum, adjacent and distant structures of the three cerebellar peduncles (superior, middle, and inferior). These structures constitute the main connection system between the cerebellum, the brainstem, and the cortex, with 70–80% of fibers providing contralateral connections and the remainder being ipsilateral (34, 41–43).

The superior cerebellar peduncle (SCP) develops between the 28^th^ week and the 6^th^ month and contains fibers converging from the dentate, globose and emboliform nuclei direct to the cerebral cortex *via* the contralateral red nucleus and the thalamus.

The middle cerebellar peduncle (MCP) develops between 42 weeks and 3 years. It consists of afferent fibers traveling from the cerebral cortex to the cerebellum, *via* the pontine nuclei. The pontocerebellar fibers have a transverse orientation in the ventral part of the pons. These fibers run laterally in the cerebello-pontine and in the cerebello-mesencephalic fissures and radiate within the white medullary body terminating in all lobules of the cerebellum, except for the nodulus and the flocculus. Fibers running anterior to the dentate nucleus are divided into a supra-dentate and infra-dentate component, the latter providing connections to the tonsillar peduncle.

The inferior cerebellar peduncle (ICP) is composed of a superficial and a deep component. It carries both afferent and efferent fiber tracts connecting the cerebellum with the vestibular system and the spinal cord, and incoming projections (climbing fibers) to the cerebellum from the inferior olive. Superficial fibers develop between 36 weeks and 4 months (outer part). They run with a centripetal direction from lateral to medial toward the cortex of the vermis. The deep component develops between 26^th^ and 36^th^ weeks of gestational age. These fibers run at the junction of the dentate nucleus with the initial portion of the SCP and direct dorso-laterally to the trigeminal nerve forming the posterior boundary of parabrachial recess between the SCP (medial) and MCP (lateral) (9, 44–51).

It is worth noting that investigation of cerebellar WM maturation has been restricted by methodological constraints, such as the three-dimensional geometry of cerebellar folia and the associated connectivity, the limited resolution of fetal MR imaging, and motion influence on the quality of exam. These barriers mainly reduce the possibility of acquiring accurate quantitative data, limiting the analysis to qualitative observation of indirect inferences from diffusion and anisotropy data. To enhance representation of fiber orientation and distribution in conventional DTI studies, Takahashi et al. applied the high-angular resolution diffusion imaging (HARDI) method to three-dimensional maturation of cerebellar connections from fetal to adult stages cerebrum. This 2014 study demonstrated that, at the earliest gestational age, pathways forming the cerebellar peduncles are already present, but pathways between deep cerebellar nuclei and the cortex are not observed until after the thirty-eighth week (40).

## The Cerebellar Anatomo-Functional Network

### Intra-Cerebellar Connectivity

The functional processing of the cerebellum is strictly related to its internal cellular organization. As previously mentioned, from superficial to deep, the cerebellar cortex is arranged into three layers: the molecular layer, the Purkinje cell layer, and the granular layer. These three cortical layers contain five main cell types, including Purkinje cells, stellate cells, basket cells, Golgi cells, and granule cells. Granule cells are excitatory, while the other cells are all inhibitory. Every granule cell receives input from mossy fibers that originate in the pontine nuclei (52).

Granule cell axons ascend to the molecular layer, where they bifurcate to form parallel fibers. Purkinje cells make up the output elements of the cerebellar cortex. Every Purkinje cell receives input from many parallel fibers and from a single climbing fiber that originates in the inferior olive. Purkinje cells provide the sole outflow from the cortex in the form of an inhibitory projection to the cerebellar and vestibular nuclei.

This architecture forms the cerebellar *micro-zones*, which represent the effective functional units. The cerebellar cortex is composed of several thousands of micro-zones. A cortical micro-zone is connected to the inferior olive and the deep cerebellar nuclei to form a cerebellar *micro-complex*. A cerebellar micro-complex can extend to include several micro-zones located in separated cerebellar regions. The micro-complexes correspond to the cerebellar *modules*. Each module is defined by its climbing fiber input coming from a specific subdivision of the inferior olivary complex which targets one or more Purkinje cells, connected to the deep cerebellar nuclei.

This configuration undergoes dramatic changes during the postnatal maturation. In fact, it has been demonstrated that the cerebellar circuit does not simply develop from a rough outline to the adult state but undergoes a series of regulated steps involving transient connections and synaptic components working together to guide the emergence of the mature cerebellar circuit (52).

### The Cerebellar Loop-Systems

**Figure 1:**
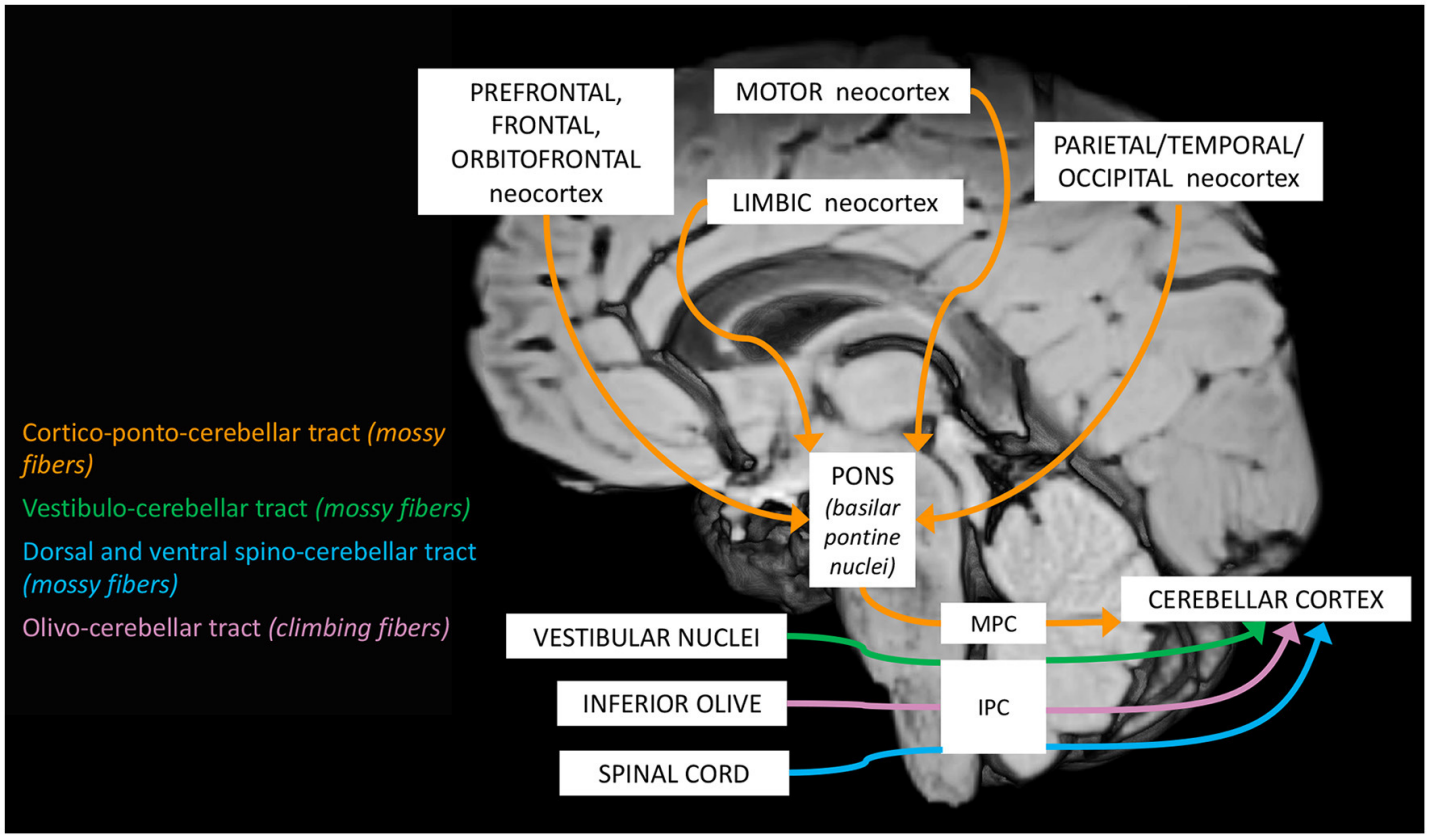
Schematic representation of the main cerebellar afferent pathways.

**Figure 2:**
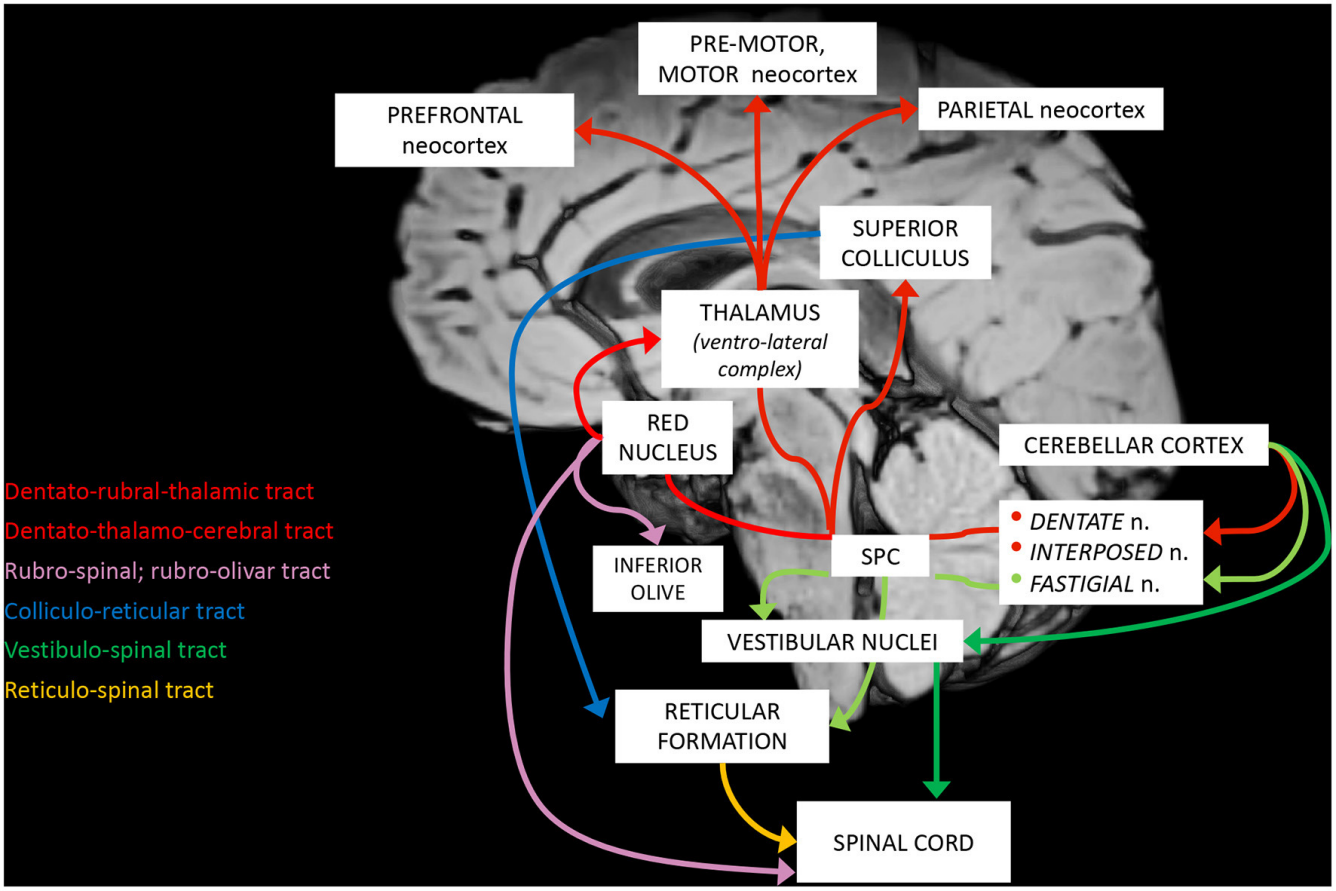
Schematic representation of the main cerebellar efferent pathways.

**Figure 3:**
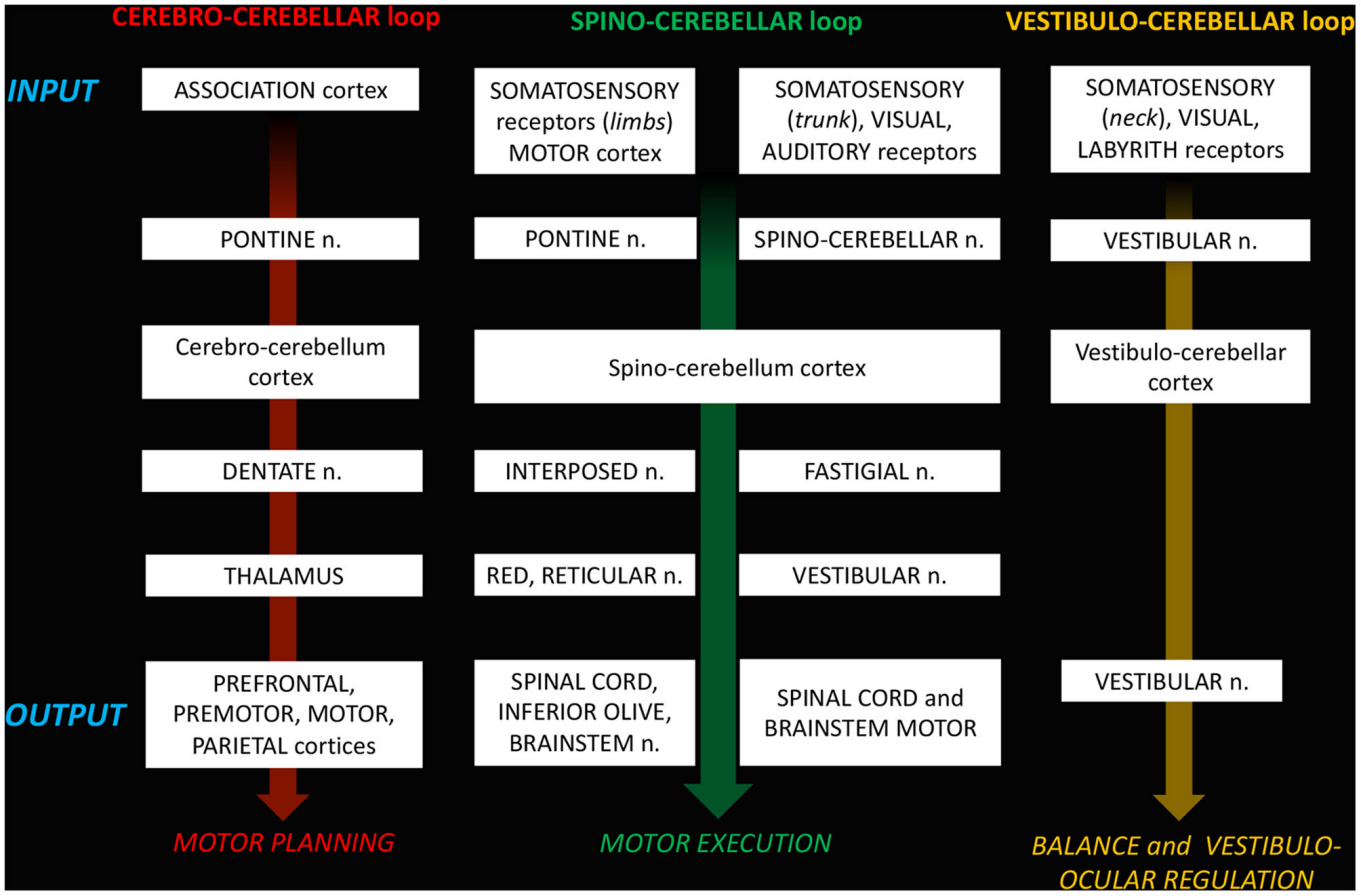
Schematic representation of the loop-organization of cerebellar circuits.

The complex maturation process described in the previous sections leads to the final setting of the cerebellar circuitry, consisting in a multichannel network of parallel channels, in which signals remain separate throughout the whole circuit, to arrange distinct loop-shaped inputs and outputs pathways (Figures 1, 2). This closed-loop organization represents the fundamental anatomo-functional architecture at the basis of both intracerebellar system and extracerebellar interactions with different cerebral, brainstem, and spinal compartments (2, 53–55) (Figure 3).

The main loops include (52, 54):

a. The *cerebro-cerebellar loop*, including inputs from frontal, prefrontal, orbitofrontal, premotor, motor cortices. These fibers converge into the pontine nuclei and reach the cerebro-cerebellum cortex, passing through the contralateral MCP. The cerebro-cerebellum is the most *lateral* cortical compartment, involving the cerebellar hemisphere and the *dentate* nucleus. From the dentate nucleus, an ascending way is direct to the contralateral red nucleus within the ventral tegmental area (*dentato-rubro-thalamic* tract) or direct to the cerebral cortex of the primary motor, premotor, supplementary, prefrontal, parietal, limbic, and temporal regions, passing through the ventrolateral nucleus of the thalamus (*dentato-thalamo-cortical* tract). The dentato-rubro-thalamic tract is classically described as a decussating pathway, ascending to the contralateral thalamus. However, recent works applying deterministic fiber tractography in healthy subjects and human brain microdissection has shown the existence of a non-decussating pathway (34). Moreover, a different topography of thalamic terminations has been demonstrated, with contralateral fibers preferentially targeting the anterior and lateral nuclei, and ipsilateral connections reaching more posterior and medial nuclei (42).
b. The *spino-cerebellar loop*, passing through the somatosensory cortex, visual and auditory receptors, the spinal cord, through the dorsal and ventral *spino-cerebellar tracts*. The spino-cerebellum corresponds to the *intermediate* compartment, including the paravermis and the *interposed* nucleus (including the *globose and emboliform nuclei*), and the *medial* compartment, involving the vermis and the *fastigial* nucleus. Efferent connections influence motor neurons and interneurons of the spinal cord and brainstem, *via* the superior colliculus, the red nucleus, the inferior olive, the reticular formation, and the vestibular nuclei.
c. the *vestibulo-cerebellum loop* partially differs from this scheme, since it projects information coming from somatosensory, visual, labyrinths receptors directly to vestibular nuclei located in the brainstem passing through the cerebellar vestibular nuclei, located within the flocculo-nodular lobe.

As a result, since the same cerebral cortical area constitutes the simultaneous target of cerebellar output and the major source of input, the cerebellum respects a strict functional segregation and never contacts the final executive neurons, but rather modulates their activity indirectly (54–56). The continuous differentiation of these cerebellar loops from childhood and adolescence to adulthood is crucial for healthy brain maturation (57).

On the other side, the same cerebellar structures may be involved in different connectivity patterns. For example, cerebellar nuclei relate to both the ventrolateral motor and non-motor thalamic nuclei. The interpositus and dorsal dentate nuclei project to the motor cortex, and the ventrolateral dentate nucleus projects to the prefrontal cortex. The dorsal part of the dentate nucleus is connected to the supplementary motor area (SMA), which in turn projects to the primary motor cortex and spinal cord. The ventral portion of the dentate nucleus projects to the pre-SMA, which is interconnected with prefrontal regions (3, 52, 58, 59).

## Cerebellum and Neurocognitive Development

### Functional Anatomy and Topography

Increasing results coming from neuroanatomic, neuroimaging, and clinical investigations conducted over the last three decades have allowed for a revision of the concept of the cerebellum as exclusively involved in motor control. On the contrary, a dense network of reciprocal connections with many supratentorial association areas is involved in many aspects of high-level neurocognitive processing (60–66). Moreover, a high degree of regional specialization of cerebellar functional topography has been demonstrated in both the adult and the pediatric population. According to this evidence, the anterior and inferior posterior cerebellum lobes are predominantly involved in sensory-motor performance. The superior posterior cerebellum is mainly associated with cognitive and social behavior functions. Emotional aspects are potentially related to the vermis (61, 62, 67–71).

Thanks to the loop system organization, the cerebellum plays a crucial modulatory role in the anatomo-functional optimization for both motor and non-motor processing within the developing brain throughout childhood and into adolescence (52).

Interestingly, the microscopic structure of the cerebellar cortex and its basic circuitry do not display significant differences in the various cerebellar lobes and lobules. In other words, the high heterogeneity of cerebellum involvement in functional processing is supported by a relatively constant structural configuration. This might relate to the strategy of improving stability, consistency, regularity, automatization of the regulation and modulation process of the supratentorial compartment, while reducing energy request (2).

MRI studies indicate that the cerebellar structure changes with age, peaking at 12 years of age in females and 15 years in males (72). However, the cerebellar development is not linear, but instead follows an asynchronous sequence. Resting state analysis revealed that more phylogenetically recent regions mature later. In fact, infants have a strong functional connectivity corresponding to the sensory-motor system, while children and adults also have associations with executive control and default mode systems, especially during the middle childhood (73). Moreover, the connectivity between the ipsilateral cerebellum and the contra-lateral primary sensory-motor cortex is stronger in adults than in children and adolescents, and vice-versa for connectivity within the local cerebellum. These findings are in favor of a *local-to-distant* development of cerebellar networks (74).

It is worth noting that several aspects of the cerebellar functional anatomy coming from animal studies need to be further clarified in humans. For example, several Authors discussed about existence and functional role of reciprocal connections between the cerebellum and the hypothalamus. After previous evidence in animal models using track-tracing and electrophysiology methods, direct cerebello-hypothalamic connections passing through the SCP have been recently described also in the human brain using fiber dissection and DTI technique (2, 32, 75, 76). Even if the functional orientation (afferent or efferent) of this pathway needs to be clarified, these results would confirm the effective participation of the cerebellum in typical hypothalamic processing, such as cardiovascular osmolarity regulation, feeding behavior, energy balance, and weight control (77, 78).

Another example concerns the existence of a cerebellar-mammillary network. Afferent connections from the lateral mammillary nucleus to vermis lobule IX (uvula) and the cerebellar anterior lobe in were described exemplars of rodents and primates, while studies performed in monkey reported different possible patterns of ipsilateral and contralateral mainly afferent projections from lateral and medial mammillary, supramammillary, and tuberomammillary nuclei to the deep cerebellar nuclei (75, 79, 80). In humans, more recent tractography analysis in healthy subjects revealed both ipsilateral and contralateral pathways between the mammillary bodies, cerebellar cortex, and dentate nucleus, reinforcing the cerebellum role in several autonomic functions, visuo-spatial orientation, and memory (81).

### The Motor Cerebellum

Growing literature, coming from tracer studies in non-human primates, human diffusion tensor imaging and functional connectivity measures, lesion studies, and electrical and magnetic stimulation studies is highlighting the anatomo-functional substrate for the implication of the cerebellum in motor function (82).

Anatomical studies demonstrated that the cerebellum output system projects to all components of the voluntary, balance and postural motor systems, through connections with both homolateral and contralateral agonist, antagonist and synergist muscles which are automatically activated during the motor learning process and most voluntary movements of daily life, including the timing of rhythmic movements (83, 84).

More recently, a strict bidirectional interplay between the cerebellum and the basal ganglia was also demonstrated in animal models. In humans, such interactions were corroborated by neuroimaging and stimulation results in neurological motor diseases associated with basal ganglia disorders, such as Parkinson's disease, writer cramp, and primary dystonia (85, 86).

Based on results of different contribution from clinical and neuroimaging research, Manto et al. summarized in a consensus paper, the large involvement of the cerebellum in several aspects of sensorimotor control, including oculomotor function, classical conditioning, motor speech, grip strength, voluntary limb movements, timing, sensorimotor synchronization, corticomotor excitability, movement-related sensory data acquisition, and interaction with the cerebrum in visuo-kinesthetic perception of hand movement, functional neuroimaging studies and magnetoencephalographic mapping of cortico-cerebellar dynamics (87).

Further crucial contribution came from clinical ataxiology. In fact, studies performed in both the pediatric and the adult population investigated the role of the cerebellum in three main categories of motor symptoms and signs related to the so-called “cerebellar motor syndrome”, including:

(i) speech deficits (dysmetria of speech, impaired motor timing, and abnormal sequencing): ataxic dysarthria, observed in lesions of both hemispheres, with a predominance for lesions on the right side; cerebellar mutism, associated with resection of midline tumor, traumatic events, strokes, or infections;
(ii) impairments of limb movements: hypermetria or hypometria dys-diadochokinesia, cerebellar tremor, isometrataxia, disorders of muscle tone, and impaired check and rebound;
(iii) abnormalities of posture and gait: ataxia of stance (increased body sway with a broad-based stance), irregular and staggering gait (87).

The cerebellar nuclei are key components of this circuitry. Each cerebellar nucleus has a somatotopic representation of the body. For example, two aspects are of special interest. The first one is related to the *interposed nucleus*, which is activated according to temporal patterns, for fine adjustments of the motor output and coordination of both single and multiple muscles. Purkinje neurons receive a massive convergence of inputs from parallel fibers, which allows to integrate the neural information coming from different sources. Their simple spikes modulate weakly during passive movements, but strongly during active movements. The activity of complex spikes following discharges constitutes a teaching signal or a motor clock signal, constituting a synchronization function of the cerebellar cortex.

The other aspect concerns the *dentate nucleus*, that is responsible for the 75% of cerebellar projections to the primary motor cortex, and for the remaining array of connections with the ventral premotor, the supplementary motor area, and with other cognitive prefrontal and posterior parietal areas (52, 88). The functional anatomy and connectivity of this structure has been revisited in a recent study by Tacyldiz et al. Based on their WM dissection technique, a compartmentalization of the DN, including the lateral major, lateral anterosuperior, posteromedial, and anteromedial areas, territorial distribution of WM connections and involvement in the SCP decussation have been carefully characterized. Fiber dissection results were consistent with data from previous functional studies. In fact, the major lateral compartment of the dentate nucleus showed both motor and nonmotor functions, the posteromedial compartment of the dentate nucleus is involved in motor functions, whereas the lateral antero-superior part and non-crossing fibers from the anteromedial compartment are related to nonmotor functions (89).

Dentate nuclear neurons fire at a permanent rate of discharge allowing the sensitivity of target structures to be tuned. This activity increases before the onset of movement and even before the firing of the motor cortex, indicating that the cerebellum is actively involved in the process of movement initiation (52).

Application of non-invasive brain stimulation techniques, such as transcranial magnetic stimulation and transdermal cortical stimulation, in both healthy subjects and cerebellar ataxic patients allowed to evaluate the role of cerebellum in motor control and learning. These functions would depend on connectivity between the cerebellum and the primary motor cortex. Intensity of cerebellar inhibitory output would correlate with quality of movement precision during reaching performance tests, and vice versa (90). On the other hand, results coming from experimental brain stimulation revealed that modulation of cerebellar activity would influence the speed of motor adaptation by decreasing cerebellar-brain inhibition (86, 91, 92).

Two circuits crucially involved in the motor aspects of cerebellum physiology are the olivo-cerebellar and rubro-cerebellar networks. Previous animal and human studies characterized the large connectivity between these structures and several cortical and subcortical areas and highlighted their participation in the extracerebellar connectivity (93).

As resumed in a consensus paper by Lang et al., the olivo-cerebellar circuit is more flexible than traditionally believed. In fact, several experimental findings showed that this system contributes to multiple aspects of the cerebellar motor control function, by generating ongoing motor commands and optimizing future motor performance by gating synaptic plasticity (94). On the other side, inferior olive denervation, associated with lesion of inhibitory projections from deep cerebellar nuclei through the central tegmental tract, would cause hypertrophy and disinhibition, altering the normal tonic firing pattern, thus likely resulting in abnormal olivo-cerebellar feedback manifesting as tremor (95).

Concerning the red nucleus (RN), lesion studies reported that rubro-cebellar circuits are compromised in several motor symptoms, including classic Holmes tremor, oculopalatal tremor, essential tremor, asynergia, adiadochokinesia, dysmetria, and non-motor manifestations, such as memory impairment, decreased verbal fluency, and intellectual fatigability (96).

These data confirmed results coming from both the animal literature and human functional studies, that provided converging evidence on the participation of the RN in planning, initiation and termination of motor tasks, but also in higher functions, such as sensory discrimination, salience detection, and executive functions (86, 97–99). The rich functional implication of the rubro-cerebellar system depends on a complex involvement of the RN in parallel and synergic networks, involving many cortical of cortical and subcortical areas, including basal ganglia, prefrontal cortex, occipital cortex, posterior hippocampus, caudal insula, thalamus, hypothalamus, left precuneus, superior temporal cortex, presupplementary motor area (preSMA) (100, 101).

Finally, observations in patients affected by the cerebellar motor syndrome indicate that the cerebellum is essential to performing accurate motor predictions and timing commands by generating *internal models*, thanks to its strict integration in large-scale brain networks. In fact, the capacity of the brain to generate predictions by integrating spatial, temporal, and environmental information is critical to performing movements with correct timing and to have adequate perceptual judgments in several tasks of daily life. Childhood is a critical period for the acquisition of this capacity. It has been demonstrated that anticipatory behaviors, especially for grasping, drawing, and postural control, are strictly related to both development of internal models for eye movements, as well as visual and proprioceptive feedback control. Training the accuracy of these internal models starts approximately at the age of 2 years and requires around seven to 10 years to mature to adult levels (102).

### The Language Cerebellum

A growing number of neuroimaging and clinical studies are highlighting the cardinal role of the cerebellum in linguistic processing *via* strong cerebello-cerebral interactions.

After the first evidence in healthy subjects in the late 1980s, several positron emission tomography studies confirmed the consistent involvement of the right posterior, lateral and inferior cerebellum during association and word generation tasks (103). A few SPECT perfusion studies highlighted the phenomenon of diaschisis, that is the reciprocal impact by a cerebellar or supratentorial lesion on the distant contralateral cerebral or cerebellar region, respectively, and provided evidence in favor of a “lateralized linguistic cerebellum” (104–110).

Introduction of fMRI technique allowed to better characterize the cerebellar language-specific activations associated with both expressive and receptive tasks, (especially at the level of lobule VI, crus I/II, and midline lobule VII of the right cerebellum), and defined the crossed anatomic interaction with the language association areas within the dominant frontal, parietal, and temporal regions (111, 112).

Moreover, it has been demonstrated that prematurity itself, cerebellar malformations or acquired injuries, involving totally or partially the cerebellar hemispheres, the vermis or the peduncles, may affect speech and language development, with varying degrees of severity, ranging from absence of language to high-level meta-linguistic disorders, according to the site and extent of the defect (113, 114).

Analysis of resting-state fMRI data in unilateral or bilateral malformation patients showed an impairment of the executive-control network involving areas strongly connected with the cerebellum through the fronto-pontine fibers (115, 116).

Lesion studies concerning both children and adult cases reported several different types of motor and non-motor language deficits after cerebellar damage, ranging from pure motor speech disorders (e.g., ataxic dysarthria) or linguistic processing impairments (e.g., mutism, conduction-like aphasia, phonemic, semantic, syntactic, lexical deficits, agrammatism, dysprosody), to high-level metalinguistic disturbances (e.g., sentence construction, word definitions, figurative language, word associations, lexical-semantic manipulation) (117–128).

These data show that the cerebellum is implicated not only in motor and planning aspects of language, but participates also in high-level non-motor linguistic processes, including speech and language perception, verbal working memory, phonological and semantic verbal fluency, syntax processing, grammar processing, as well as reading and writing (110, 129).

Investigation of the neuroanatomical substrate for this functional complexity revealed that a close connection between the cerebellum and the supratentorial motor, paralimbic, and association cortices is established by a dense network of cortico-ponto-cerebellar and cerebello-thalamo-cortical loops. Moreover, functional connectivity analyses demonstrated that, as for motor function, a topographical organization exists also for cognitive and language processing, with representation of the muscles of articulation located in medial lobule VI, whereas conceptual elements of language are mostly represented within the right posterior-lateral regions of lobules VI and VII (3, 61, 71, 128).

As for motor function, the network between the cerebellum and different supratentorial centers reinforces the concept of a regulatory role of the cerebellum in monitoring and coordinating cortical functions. This phenomenon, called “universal cerebellar transform” allows the cerebellum to compare predictions related to internal speech or subvocal rehearsal, neural representation of the external world (called “internal models”), with the incoming feedback from the cerebrum. The internal models are used to create, manipulate, and predict mental representations, by comparing them with the prefrontal and temporo-parietal cortex to optimize cognitive and language performance (71, 110).

### The Cognitive, Emotional and Behavior Cerebellum

The third important aspect of cerebellum functional anatomy concerns its contribution to neurocognitive processing. Also in this field, many works provided consistent neuroimaging and clinical data on the strict interactions between the cerebellum and different cortical and subcortical territories involved in cognition, executive, spatial, working memory, emotional, and behavior processing and social domains (61, 71, 109, 129).

The anatomo-functional network related to cognitive and emotional domains develop at a later stage, according to emergence of long-range cerebro-cerebellar connectivity. Moreover, the development of intrinsic connectivity extends beyond the first years of life into late adolescence, consistent with changes in executive function, social interactions, and risk-taking behavior (130, 131).

Clinically, alteration of executive functioning has been associated to different etiologies occurring during both childhood and adulthood, including congenital malformations (cerebellar or vermian hypoplasia, atrophy, or dysplasia), and acquired cerebellar damage, such as viral inflammatory lesion or tumors. These diseases adversely impact the development of both cerebellar gray volume and the integrity of distant projections to basal ganglia and non-motor cortical regions. Seriousness of clinical consequences depends on both extension of structural defect and timing of lesion event, ranging from mild neuropsychologic delay to severe and long-lasting intellectual disability and neuropsychiatric disorders, such as schizophrenia, Tourette's syndrome, autism, attention deficit disorder, and addiction (132).

Concerning emotional processing, early electrophysiological and anatomical studies in humans and animals that revealed that stimulation of specific cerebellar territories, such as the dentate nucleus and the SCP, evocated different emotional responses or mood modification after chronical stimulation, and demonstrated reciprocal connections with limbic structures (133–137).

Following functional connectivity studies demonstrated that the vermis is a crucial region functionally connected to limbic structures allowing for emotional regulation (through the amygdala), emotional memory (through the hippocampus), and autonomic and cognitive components related to emotional experience. In fact, the vermis has an early-stage pattern of maturation, and it does not show significant changes in volume during childhood and adolescence. This evidence confirms that this phylogenetically “older” part of the cerebellum is recruited during primary emotional processing (138–140).

Social cognition is defined as the set of mental processes required to understand, generate, and regulate social behavior and interpersonal interactions. Social cognition is strictly related to the mentalizing, which consists in the ability to understand the mental state of oneself or others underlying overt behavior (64, 71). Previous researchers identified the brain regions most involved in the “mentalizing network”, including the temporoparietal junction, the precuneus, the amygdala, the anterior temporal lobe, the occipital gyrus, the fusiform gyrus, the inferior frontal gyrus, and the medial prefrontal cortex (141–145).

Although participation of the cerebellum to this circuitry received less attention in the past, more recent resting state fMRI studies indicated a functional overlap between the social cognition network of the cerebellum and the mentalizing network of the cerebrum, namely the default-mode network (DMN), which involves a set of brain regions such as the posterior cingulate cortex, the precuneus, the lateral parietal/angular gyrus, the medial prefrontal cortex, the superior frontal gyrus, and the temporoparietal junction (146).

This finding was also reported in clinical studies on diseases typically characterized by an impairment in social mentalizing, such as autism spectrum disorder (147). Otherwise, the possibility that cerebellar damage might cause social cognition or mentalizing deficits would depend on the developmental stage during which the injury occurs, the location of injury, and testing modality (64). According to this model, the role of the cerebellum would be to modulate the activity of default regions by detecting errors between predicted output and current behavior through construction of internal models. In case of mismatch, the feedback error signal would be sent back to the same cerebral regions (146).

Other Authors investigated the specific cerebellar zones mostly implicated in mentalizing processes in association with the human DMN (139, 148–155). For example, a meta-analysis of functional connectivity studies revealed a strong recruitment of posterolateral cerebellar hemisphere corresponding to crus I/II in bilateral mentalizing cerebral regions, such as the left and right temporoparietal junctions, the precuneus, and the medial prefrontal cortex. Within this circuit, the medial prefrontal cortex and the right temporo-parietal junction converge on the right posterior cerebellum and then back to the left temporo-parietal junction, which in turn receives connectivity from the dorsal medial prefrontal cortex and the right temporo-parietal junction. Interestingly, it was shown that cerebello-cerebral connections are not directly linked in both directions, as expected, but have possibly divergent patterns of information communication with other regions during social cognition processing (150).

More recently, Metoki et al. applied a multimodal neuroimaging approach, including functional mapping, effective connectivity, and probabilistic tractography, to investigate the structural and functional role of the cerebellum in the mentalizing network. In addition to confirming that also mentalizing has a domain-specific function, the authors identified stronger effective connections from the left posterior cerebellar hemisphere to the right cerebral mentalizing areas through cerebello-thalamo-cortical and cortico-ponto-cerebellar connections to right cerebral areas mostly involved in mentalizing processing (7, 64, 149).

Finally, electrophysiological, pharmacological and immunohistochemical research studies investigated how different neurotransmitter systems, such as noradrenergic, cholinergic or dopaminergic system, can modulate the synaptic transmission and thereby influence the functional output through activation of specific cerebellar receptor subtypes (156–166).

Norepinephrine responsive cells were identified within both the cerebellar cortex and the cerebellar nuclei (156). Moreover, recent studies in rat have shown that GABAergic synapses of cerebellar interneurons and Purkinje cells are positively regulated by norepinephrine. The overall effect of the noradrenergic afferent input to the cerebellar cortex reinforces GABAergic inhibitory influence from the basket cells to the output signals generated by Purkinje cells. The subsequent modulation of GABAergic transmission at synapses between input and output cells appears to play a critical role in motor coordination associated with the cerebellar connection with the somatosensory cortex (167).

The cholinergic system has been shown to have an important modulatory effect on synaptic transmission, especially at the level of climbing and mossy fibers, and in regulating release of other neurotransmitters. This (plastic) effect would influence the rapid intracerebellar and cerebro-cerebellar causal connectivity, so conditioning the final functional cerebellar outputs during non-motor cognitive task. Moreover, modulation of neuronal excitability and/or synaptic responses would have a significant role during cerebellar development, by facilitating neuronal maturation, differentiation, adaptation, and survival. On the other hand, cholinergic dysfunctions were shown to be involved in the pathogenesis of cognitive impairments and other neurological disorders, such as cerebellar ataxia, autism, and Parkinsonism (157).

Concerning the dopaminergic system, the cerebellum was traditionally not considered an elective dopaminergic region. However, lesion studies have shown the involvement of cerebellum in dopamine deficit-related neurological and psychiatric disorders, such as Parkinson's disease, schizophrenia, autism spectrum disorders, and drug addiction (158–161). Moreover, data coming from invasive studies in animal and tractography observation in humans revealed relevant patterns of intracerebellar dopaminergic connectivity, such as between the DN and the cerebellar cortex, and extrinsic interconnections between the cerebellum and traditional dopaminergic areas of the brain, including the midbrain, the neostriatum, the neocortex (162–165). These connections could represent part of the cerebellar projective circuits which allow the cerebellum to contribute to motor and cognitive functions (164, 166).

Interestingly, the role of cerebellum in different neurotransmitter systems may have implication for innovative non-invasive treatments (e.g., electrical, or magnetic stimulations) for several neurological and mental disorders (166).

### The Visual and Attention Cerebellum

Previous research from animal models and patients with developmental abnormality (e.g., autism) or acquired injury (e.g., tumor and stroke) showed that damage to the cerebellum was associated to slowed spatial attention orienting (168–170).

Following fMRI studies in healthy subjects confirmed these observations and investigated the structural substrate underlying the cerebellum participation to a network of cortical regions, including the posterior parietal cortex, the frontal eye fields, and the dorsolateral prefrontal cortex (the so called, dorsal attention network or fronto-parietal network) that support sustained attention and working memory (171). More specifically, analysis of functional connectivity in intact brains demonstrated that the lobule VI of the left cerebellar hemisphere in both covert attention and saccadic eye movements (172).

Other Authors identified a strong functional correlation between cortical nodes forming the dorsal attention network and cerebellar lobules VIIb and VIIIa within the posterior cerebellum during visual attention and working memory tasks. Moreover, different functional patterns between the dorsomedial and ventrolateral regions of lobule VIIb/VIIIa were also found in relation with visuospatial representations and load-dependent visual working memory processing, respectively (173–175).

More recently, a comprehensive mapping of the cerebellar visuospatial organization based on a retinotopy dataset collected in 181 participants revealed a large distribution of signals involving the vermis and the lobules VIIb and VIIIb. In particular, connections between retinotopically organized cortical regions of lobule VIIb with the intraparietal sulcus confirmed involvement of this structure in spatial cognition, including attention and working-memory-related processes. The lobule VIIIb was found to be related to the dorsal and the ventral attention networks and to the somato-motor network, suggesting an integrative role for this region for both attentional and motor processes. In summary, these data are in favor of participation of the cerebellum in attentional mechanisms by prediction of dynamic perceptual events and integration of visuospatial information for the guidance of effector movements (176).

## Implications

The crucial involvement of the cerebellum in sensory-motor and higher cerebral functions may correlate with a variety of clinical consequences in case of pathological processes, including developmental disorders, malformations, stroke, and tumors or iatrogenic causes (10). These conditions may alter the normal anatomo-functional configuration of gray and WM components forming the cerebellar connectome, at both the local and the remote level of the network.

Considering WM structures specifically, direct damage or disconnection of input or output connections with different cerebral supratentorial eloquent regions passing through the cerebellar peduncles should be considered. In fact, anatomo-clinical studies showed that lesions of the SCP cause ipsilateral intention tremor, dysmetria, and decomposition of movement; lesions of the MCP are associated with homolateral hypotonia, ataxia, and dysmetria during voluntary movement; injury to the ICP induces disturbances of equilibrium with truncal ataxia and staggering gait (35, 36, 44).

Concerning neuro-oncology, previous studies indicated that cerebellum undergoes structural and functional alterations, especially concerning language function, induced by supratentorial high-grade and low-grade gliomas, confirming the role of cerebellar system in lesion induced plasticity process (177, 178).

It is worth noting that a majority of previous studies evaluated the local and distant effects of posterior fossa tumors after treatments, especially surgery and radiotherapy, while only a few authors focused on the direct effects of cerebellar tumors on WM structures. Currently, the main evidence on tumor-related alteration of WM integrity concerns the cerebello-thalamo-cerebral tracts. Significant impairment of this pathway was shown, especially in patients with diagnosis of posterior fossa medulloblastoma, large tumors with bilateral extension that cross the midline, and left-handed subjects. In these cases, a significant correlation between the overall damage of the cerebro-cerebellar connectivity and extent of ataxia and fine motor dysfunction has been demonstrated (179).

Regarding the possible effects of surgery and other treatments, a perfect example is posterior fossa syndrome (PFS), which includes ataxia, muscular hypotonia, hemiparesis or tetraparesis, and possible cranial nerve deficits. This condition is frequently associated with cerebellar mutism syndrome, which is characterized by diminished psychological impulsion associated with inhibition of speech output. These well-recognized complications occur at a high rate in children (10 to 40%) who undergo posterior fossa surgery for both malignant and benign tumors (180–185).

In the past, different factors, including brain stem compression, hydrocephalus, and damage to the inferior cerebellar vermis were considered responsible for PFS (183, 186). More recent large scale-studies revealed that the pathogenesis of PFS depends on a more complex involvement of cerebellar connection systems, including the dentate nucleus and the superior cerebellar peduncle of fronto-cerebellar pathways, independent of chemotherapy or radiation (187–191).

In addition, a significant association between cerebellar tumors and neurocognitive performance, especially working memory, was also demonstrated when a global functional depression of cerebral cortical activity (with a predilection to frontal regions) was found in patients with PFS, related to a decreased integrity of WM pathways and a diaschisis-related mechanism (192–194).

Functional-MRI studies contributed to further characterizing these aspects. A recent study found that cognitive disturbances in pediatric infra-tentorial tumor survivors were related to abnormalities in regional neural synchrony, leading to reorganization of network topology and, consequently, to disconnection of damaged regions and multiple extracerebellar functional brain networks (195). In the largest lesion mapping study to date, the cognitive and affective symptoms following pediatric cerebellar tumor resection were evaluated in 195 pediatric patients. Comparison between resting-state functional connectivity MRI patterns of pathological cases and normal subjects confirmed the role of disrupted cerebro-cerebellar connectivity, particularly involving a network including the fastigial and dentate nuclei, the thalamic mediodorsal nucleus, and projections to the limbic system (anterior cingulate, medial, lateral prefrontal, and orbitofrontal cortices) (196).

Interestingly, it was reported that seriousness of PFS depends also on patient-specific presurgical status. A significant decrease in preoperative neural connectivity involving the corpus callosum, the right cortico-thalamic pathway, and the right cortico-striatal pathway was found in children who developed a post-operative PFS, compared with those who did not (197).

Finally, a positive correlation between the cerebellar volume of output structures and integrity of WM output was shown. Impairment of this correlation, also called trans-neural degeneration, may produce different consequences depending on type of treatment. Without radiation, the primary mechanism of injury is cerebellar tumor growth, resection, and hydrocephalus. Therefore, the lesion involves the most proximal connection (cerebellar-rubral pathway). In contrast, the survivor group treated with radiation may have most extended radiation-induced demyelination of the thalamic-frontal portion of the pathway, based on a strong correlation with volume loss in the cerebellum, the red nucleus, the thalamus, and the frontal lobe (198).

### Neurosurgical Implications

Progressive advancement in knowledge on the cerebellar connectome may have important implications for the neurosurgical community, especially regarding management of posterior fossa tumors and vascular malformations (199, 200).

Complexity of the anatomo-functional organization of the intra-cerebellar and the extra-cerebellar circuitry is directly related to the need for careful awareness of surgically related neurological consequences at short and long term. For this reason, the authors of a recent systematic review emphasized that *cerebellar functional anatomy should receive similar awareness during infratentorial surgery as during supratentorial resections* (201).

In this perspective, two aspects might be of special interest to improve the quality of surgical results and functional outputs. First, an accurate understanding of the three-dimensional functional anatomy and topography of cerebellar WM is crucial to select the most appropriate approach, especially in the case of approaches involving the fourth ventricle (44). Fascinating didactic dissection and tractography studies have been devoted to describing the surgical anatomy of the human cerebellum and to discussing implications of main eloquent cerebellar nuclei and cerebellar peduncles in most common surgical routes (192, 202–205).

In fact, dissection studies show that all the cerebellar peduncles converge on the lateral wall and roof of the fourth ventricle. In particular, the ICP and SCP are more likely to be injured during procedures within the fourth ventricle because they abut directly on the ventricular surface. On the other hand, the MCP would be more susceptible to injury when approaching the cerebellopontine angle, where it forms a major part of the cisternal surface of the ventricular wall (36).

In the case of approaches to intrinsic peduncular lesions, it is recommended to perform the incision on the dorsal surface of the MCP following a parallel and longitudinal direction to respect the pontocerebellar fibers. If resection involves deeper ponto-cerebellar fibers, the anatomical boundaries to be respected include the corticospinal tract ventrally, the trigeminal nerve caudally and the medial and lateral lemnisci posteriorly in the tegmental pars of the pons (206). Following these considerations, previous authors have analyzed the main anatomical surgical landmarks to be respected and compared the different possible approaches according to lesion location and extension (192, 203–206).

The second consideration is that development and systematic application of accurate neuromonitoring techniques is of paramount importance for preserving the main components of the network. However, as revealed by a recent review, despite the consolidated experience of intraoperative monitoring during brainstem, fourth ventricle, and skull base surgery, specific neurophysiological data on the cerebellum are lacking, due to the polysynaptic organization of the cerebro-cerebellar circuitry and, in pediatric cases, to the immaturity of nervous structures (88).

Consequently, apart from adoption of basic technical precautions, such as accurate neuronavigation guidance and avoidance of mechanical and thermal injury, for now the possibility of improving safety of intracerebellar surgery seems to rely on the development of other methods able to predict the postoperative outcome (201).

These include, for example: (i) computation of probabilistic connectivity atlas based on segmentation of the cerebellar peduncles, allowing for comparison of WM integrity between patients and controls diffusion maps (207, 208); (ii) application of transcranial magnetic stimulation (TMS) technique to provide either negative or positive inhibitory or facilitatory modulation of the primary motor cortex output using an appropriate cerebellar stimulus to deduce injury to the dentato-thalamic-cortical circuit and predict postsurgical development of cerebellar mutism (209); (iii) development and validation of reliable procedures for neurophysiological monitoring of the cerebello-cortical connectivity. Preliminary results in this field showed the feasibility of reproducing extra-operative conditioning-test paradigm previously adopted for TMS (210).

## Conclusions

Modern neuroscience has significantly progressed toward a more realistic characterization of cerebellar functional anatomy. A growing number of studies have confirmed that the cerebellum is involved in several networks with both short-range and long-range disposition. This organization results from a continuous process of anatomo-functional refinement continuing from childhood to adulthood, allowing for integration between intra-cerebellar connections and supratentorial circuits.

In parallel with the closed loop structural configuration, the dogma of the cerebellum being merely responsible for motor coordination has been definitively repudiated, in favor of a more complex functional role that also includes emotional and social processing.

This evidence should not be limited just to theoretical speculation, but may have important implications for neurosurgical practice, especially for management of diseases harboring the posterior fossa. In fact, application of multimodal analysis for the pre-operative and post-operative assessment of the cerebello-cerebral networks might provide crucial insights allowing for a better understanding of the effects of a pathological process and of surgery itself and optimizing the quality of intraoperative approaches and rehabilitation strategies.

In this context, some challenging yet promising topics could be mentioned for future research. For example, the role of age, of specific cerebellar sub-regions, and of plasticity mechanisms in normal and pathological motor and cognitive outcomes, especially during critical periods following early life stages should be further highlighted. Moreover, identification of specific biomarkers will allow for the development of reliable probabilistic atlases based on different convergent factors, such as the presurgical cerebellar lesion characteristic, the relationships between the lesion type and location, the cerebellar outflow, the supratentorial cortical activity, and the possible clinical deficits. Finally, all this information might contribute to stratifying the postoperative risk, so improving the quality of communication to patients and families and optimizing both presurgical planning and intraoperative management.

## Author Contributions

All authors have given substantial contributions to the conception, the draft, and the final approval of the manuscript.

## Funding

This research was funded by the Ministero della Salute-Ricerca Corrente to Ospedale Pediatrico Bambino Gesu', IRCCS.

## Conflict of Interest

The authors declare that the research was conducted in the absence of any commercial or financial relationships that could be construed as a potential conflict of interest.

## Publisher's Note

All claims expressed in this article are solely those of the authors and do not necessarily represent those of their affiliated organizations, or those of the publisher, the editors and the reviewers. Any product that may be evaluated in this article, or claim that may be made by its manufacturer, is not guaranteed or endorsed by the publisher.
